# Resveratrol and Its Metabolite as Potential Allosteric Regulators of Monoamine Oxidase A Activity in the Brain and Liver Under Chronic Predator Stress

**DOI:** 10.3390/biomedicines13051196

**Published:** 2025-05-14

**Authors:** Jurica Novak, Olga B. Tseilikman, Vladislav A. Shatilov, Maxim S. Zhukov, Vadim A. Shevyrin, Zuhra R. Khismatullina, Albina M. Fedorova, Georgiy N. Patrikyan, Timur L. Khaibullin, Vadim E. Tseilikman

**Affiliations:** 1 Centre for Informatics and Computing, Ruđer Bošković Institute, Bijenička Cesta 54, 10000 Zagreb, Croatia; 2 Faculty of Fundamental Medicine, Chelyabinsk State University, 454001 Chelyabinsk, Russia; 3 Chemical Technology Institute, Ural Federal University Named after the First President of Russia B. N. Yeltsin, 620062 Yekaterinburg, Russia; 4 Institute of Nature and Man, Ufa University of Science and Technology, 450076 Ufa, Russia; 5 Scientific and Educational Center ‘Biomedical Technologies’, School of Medical Biology, South Ural State University, 454080 Chelyabinsk, Russia; 6 Zelman Faculty of Medicine and Psychology, Novosibirsk State University, 630090 Novosibirsk, Russia

**Keywords:** resveratrol, monoamine oxidase, serotonin, *trans*-resveratrol-3-*O*-glucuronide, allostery

## Abstract

**Background**: Resveratrol has been shown to modulate stress-related anxiety by reducing brain monoamine oxidase A (MAO-A) activity. However, the molecular mechanism underlying this neurochemical effect remains unknown. In this study, we employed in silico approaches to investigate the binding affinity of resveratrol and its predominant blood metabolite, resveratrol glucuronide, to specific sites on MAO-A. **Methods**: For the in silico analysis, we employed molecular docking and molecular dynamics simulations. Within the liver–brain axis, we investigated the role of hepatic MAO-A in the development of anxiety. The activity of whole-brain MAO-A was compared with its activity in specific brain regions, including the amygdala, hippocampus, and prefrontal cortex. **Results**: Our findings suggest the presence of an allosteric site on the enzyme that accommodates these compounds. Furthermore, in vivo experiments demonstrated that high-dose resveratrol suppresses MAO activity not only in the brain but also in the liver of stress-exposed rats. The in vivo results are interpreted in the context of an allosteric site on MAO-A in both the brain and liver, which may mediate the interaction with resveratrol and its metabolite. **Conclusions**: The primary outcomes of the study include the identification of the role of hepatic MAO-A in the development of anxiety-like behavior, as well as the determination of resveratrol dose ranges at which it functions as an allosteric modulator of MAO-A activity.

## 1. Introduction

**Figure 1:**
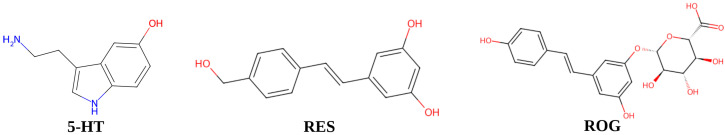
Two-dimensional structures of serotonin (5-HT), resveratrol (RES), and *trans*-resveratrol-3-*O*-glucuronide (ROG).

Resveratrol (*trans*-3,4′,5-trihydroxystilbene) (Figure 1) is a naturally occurring phenolic compound belonging to the stilbene family, predominantly found in various plant sources, including grape skins, berries, cocoa, and nuts [1,2,3]. Its levels in plants often increase in response to various environmental stressors, such as ultraviolet radiation, ozone exposure, and pathogen attacks [4,5]. Resveratrol (RES) has been recognized as one of the most promising chemopreventive agents against cancer. In addition, it exhibits antidiabetic, antiviral, cardioprotective, anti-inflammatory, and neuroprotective properties. Notably, its neuroprotective effects are largely attributed to its ability to enhance neuroplasticity [6,7,8].

Due to its neuroprotective and antioxidant properties, RES has demonstrated therapeutic potential in neurodegenerative disorders as well as in the correction of stress-related behavioral disorders [9,10]. Recent studies from our group have established the efficacy of RES in alleviating anxiety disorders induced by chronic predator stress, an experimental model of post-traumatic stress disorder (PTSD) [11,12]. These findings identified monoamine oxidases (MAOs) in the brain as key molecular targets of RES. Our results further revealed that stress-induced anxiety correlates with increased MAO-A activity, whereas RES administration effectively reduced MAO-A activity in parallel with behavioral improvements. Importantly, the anxiolytic effects of RES exceeded those of selective serotonin reuptake inhibitors (SSRIs), which are currently considered the first-line pharmacological treatment for PTSD. Notably, RES shares a common target with SSRIs—the serotonin transporter (SERT)—which is responsible for serotonin reuptake.

Despite its remarkable therapeutic potential, the clinical application of RES remains challenging due to its rapid metabolism and poor bioavailability [13,14]. Pharmacokinetic studies indicate significant losses of RES during absorption, with only a small fraction of the compound being absorbed through the intestinal epithelium without undergoing metabolism. Experiments on an isolated rat intestinal model have shown that 96.5% ± 4.6% of the absorbed RES was detected as its glucuronide conjugate, highlighting its susceptibility to glucuronidation during transport across the jejunal epithelium [13].

Interestingly, *trans*-resveratrol-3-*O*-glucuronide (ROG) itself exhibits biological activity, including well-documented anticancer properties [15,16,17]. In our studies, a correlation was observed between the levels of ROG in the blood of stressed animals and anxiety-related behavioral parameters in the elevated plus maze test, a widely used model for assessing anxiety in chronic predator stress [18]. However, the precise mechanisms underlying the behavioral effects of ROG under chronic stress conditions remain unknown.

Traditionally, the relationship between brain MAO-A and the regulation of behavioral activity has been the primary focus of research, with particular emphasis on the enzyme’s ability to metabolize monoamine neurotransmitters. However, the potential contribution of hepatic MAO-A to behavioral regulation has been largely overlooked. In the liver, MAO-A is involved in the oxidative deamination of trace amines of intestinal origin that reach the liver via the portal circulation. To date, the therapeutic effects of resveratrol on anxiety disorders have not been associated with its modulation of MAO-A activity in peripheral organs. Furthermore, paradoxical findings—such as resveratrol’s ability to reduce MAO-A activity despite an upregulation of its gene expression—remain insufficiently addressed.

Given that MAO-A has been identified as a key target of RES, we conducted an in silico comparative analysis to evaluate the binding interactions of RES and ROG with MAO-A. The computational results were further correlated with the experimental findings on RES and ROG levels in stressed animals and their impact on MAO-A activity.

## 2. Materials and Methods

### 2.1. Animals

Experiments were conducted on male Wistar rats, weighing 210–230 g and three months of age at the onset of the study. The use of males was justified by the fact that applying the same experimental protocol to females would require additional time to synchronize the estrous cycle. In this study, male Wistar rats were housed in individually ventilated cages, with each enclosure accommodating three to four animals. The rodents had unrestricted access to tap water and a nutritionally balanced diet (Beaphar Care Plus Rat Food, Raalte, The Netherlands). Environmental conditions within the vivarium were carefully regulated, maintaining a temperature range of 22–25 °C and a relative humidity of 55%. The light–dark cycle was set to 12:12 h, with illumination commencing at 07:00 and concluding at 19:00.

### 2.2. Chronic Predator Stress Paradigm

In this study, a chronic predator stress (PS) paradigm was employed. While cat urine has been conventionally used as a predator odor source in post-traumatic stress disorder (PTSD) research, its efficacy in eliciting an immediate anxiety response following prolonged stress exposure has been limited. Prior research demonstrated that prolonged exposure to cat odor led to a diminished sensitivity to this stimulus in stressed rats by the conclusion of the PS paradigm. Furthermore, time-dependent sensitization—recognized as a key feature of PTSD—only became evident two weeks after the exposure period. To provoke an immediate behavioral response characterized by acute stress-induced anxiety, cat urine was replaced with fox urine, which exhibits a stronger anxiogenic effect.

### 2.3. Fox Urine Collection and Application

Urine was obtained from sexually mature males of domesticated silver-black foxes (*Vulpes vulpes*). Collection took place during the autumn season from multiple individuals, after which the samples were aliquoted and stored at −18 °C for a maximum duration of one month. Prior to use, the urine was thawed immediately. To deliver the stressor, 100 μL of urine was deposited onto a cotton pad and placed within a plastic Petri dish covered by a nylon mesh to allow the release of volatile compounds. The Petri dish was positioned inside the animals’ home cages for a duration of 10 min each day across a 10-day period, starting on the fifth day of the experiment.

### 2.4. Timeline of Predator Stress Exposure, Resveratrol Treatment, and Plasma Resveratrol Concentration Assessment

To evaluate the effects of RES treatment on anxiety-like behavior and monoamine oxidase A (MAO-A) activity in the brain and liver, rats were subjected to a 10-day predator stress (PS) paradigm. The animals were assigned to the following experimental groups:

1. Control (*n* = 7): Rats received vehicle treatment for 10 consecutive days without exposure to predator stress.
2. PS (*n* = 7): Rats were subjected to chronic predator stress.
3. RES + PS 20 mg/kg (*n* = 7): Rats were administered resveratrol (20 mg/kg) via intraperitoneal injection one hour prior to each predator stress exposure.
4. RES + PS 50 mg/kg (*n* = 7): Rats were administered resveratrol (50 mg/kg) via intraperitoneal injection one hour prior to each predator stress exposure.
5. RES + PS 100 mg/kg (*n* = 7): Rats were administered resveratrol (100 mg/kg) via intraperitoneal injection one hour prior to each predator stress exposure.

The rationale for administering RES one hour prior to the onset of stressor exposure was based on the brief duration of a single stressor session. It was essential for RES to be present in the system at the time of stressor exposure. The PS-exposed animals were divided into two groups: one received daily intraperitoneal injections of RES at doses of 20, 50, or 100 mg/kg (“PS + RES” group), while the other received vehicle only (“PS” group). Control animals were also administered vehicle injections.

*Trans*-resveratrol was obtained from Sigma Aldrich Ltd. (St. Louis, MO, USA). Daily intraperitoneal injections of RES were administered over the first 10 days of the experiment. Fresh solutions were prepared on a weekly basis and stored at room temperature until use. RES was dissolved in 99% DMSO to achieve a final injection volume of 1 mL/kg of body weight, corresponding to a dosage of 20, 50, and 100 mg/kg [18].

Plasma concentrations of RES and its primary metabolite, ROG, were quantified in the “PS + RES” group. Correlations were examined between RES and ROG levels and various behavioral parameters, as well as MAO-A activity in the brain and liver.

### 2.5. Behavioral Testing

Anxiety-like behavior was assessed using the elevated plus maze (EPM) test, employing a standard apparatus (model TS0502-R3, OpenScience, Russia; http://www.openscience.ru/index.php?page=ts&item=002 (accessed on 9 May 2025)). The EPM consisted of two open and two closed arms (arm length: 0.5 m; arm width: 0.14 m) elevated 0.55 m above the floor, with the height of the closed arm walls measuring 0.3 m and the side rails of the open arms measuring 0.01 m [19]. At the beginning of each trial, animals were placed in the central platform facing an open arm. An entry into an arm was recorded when all four paws of the rat were within the arm. Behavioral tracking and analysis were performed using the 3D animal tracking system “EthoStudio” (http://ethostudio.com/new/en/about/ (accessed on 9 May 2025)). The test was performed over a 10-minute period. The rationale for selecting the behavioral tests was based on their established suitability for assessing anxiety levels and fear responses. To minimize potential bias, control and experimental groups were assessed concurrently under blinded conditions.

Key metrics for behavioral assessment included:

- Frequency of entries into the open and closed arms of the EPM;
- Duration of time spent within the open and closed arms.

Following the completion of each testing session, the surface of the EPM or the open field arena was thoroughly wiped with gauze soaked in ethanol to eliminate any residual traces left by the previous animal.

### 2.6. Blood and Tissue Collection and Storage

The rats were sacrificed by an overdose of diethyl ether, decapitated, and blood was collected for further analysis. During necropsy, samples of blood and liver tissue were collected from the rats. The blood was processed by centrifugation to isolate plasma, which was then transferred into Eppendorf tubes and stored at −70 °C. Liver tissue was preserved in two forms: one part was fixed in 10% buffered formalin for histopathological examination, while the remaining tissue was rapidly frozen in liquid nitrogen and stored at −70 °C for subsequent biochemical analysis.

Brain and liver tissues were rapidly frozen in liquid nitrogen and stored at −70 °C for biochemical investigations. The hippocampus, prefrontal cortex, and amygdala were dissected from freshly excised brains cooled on ice, based on anatomical landmarks defined in the Paxinos and Watson atlas [20]. These brain regions were immediately frozen in liquid nitrogen and stored at −70 °C for neurochemical analysis, which was conducted within seven days of tissue collection.

### 2.7. Quantification of RES and RES-O-Glucuronide in Rat Plasma

The concentrations of RES and its metabolite, ROG, in rat plasma were determined using high-performance liquid chromatography (HPLC) with a diode array ultraviolet (UV) detector (Agilent 1260 Infinity II, Agilent Technologies, Santa Clara, CA, USA). The separation process utilized a gradient elution approach on a Poroshell 120 EC-C_18_ reversed-phase column (3.0 mm × 100 mm × 2.7 μm; Agilent Technologies, 695975-302), coupled with a 5 mm guard column for system protection. The mobile phase comprised two eluents: solvent A (aqueous 0.1% *v*/*v* acetic acid) and solvent B (methanol containing 0.1% *v*/*v* acetic acid). A linear gradient elevated solvent B concentration from 15% to 100% over 14.3 min, and maintained at 100% for an additional 1.7 min. Operational parameters included a 0.7 mL/min flow rate and column thermostatting at 30 °C. Analyses employed 5 μL injections with ultraviolet detection configured at 304 nm.

Calibration standards were generated through spiking 50 μL of a methanol-dissolved RES working solution (Sigma-Aldrich, St. Louis, MO, USA, 98% purity) into 150 μL portions of blank plasma. This procedure yielded plasma calibration standards spanning 0.05–20.0 μg/mL RES concentrations. For sample processing, 200 μL of either plasma or prepared standards were aliquoted into 2.0 mL centrifugation vials. Each vial received 50 μL of pterostilbene internal standard solution (Sigma-Aldrich, St. Louis, MO, USA, 97% purity) at 20 μg/mL. Following 15 s vortex agitation, 0.8 mL of acetonitrile was introduced, and the mixture was vortex-mixed again for an additional 15 s. Samples were processed by centrifugation at 10,000 rpm for 15 min using a Thermo ST16R unit (Thermo Scientific, Waltham, MA, USA). Supernatants were harvested, transferred to fresh tubes, and dried under nitrogen gas at 45 °C via an NDK200 concentrator (Hangzhou MIU Instruments Co., Hangzhou, China). Dried residues were resolubilized in 0.2 mL of methanol:water (1:1 *v*/*v*), transferred to autosampler vials, and subjected to analysis.

Quantification of RES plasma levels utilized a calibration curve derived from the relationship between analyte-to-internal standard peak area ratios and corresponding analyte concentrations. ROG concentrations were calculated using the RES calibration framework, presuming equivalent detector responses between the two analytes. Identification of RES and pterostilbene chromatographic peaks was verified through retention time alignment and UV spectral matching (200–400 nm range) against reference standards.

ROG characterization was performed using an Agilent 6545 Q-TOF LC-MS system (Agilent Technologies, Santa Clara, CA, USA) with negative-ion electrospray ionization. Chromatographic parameters remained consistent with previously outlined separation conditions. Mass analysis detected a deprotonated molecular ion [M – H]^−^ at *m*/*z* 403.1036 (Δ = 0.42 ppm). Under collision-induced dissociation parameters, the MS/MS spectrum displayed elimination of a glucuronic acid moiety (176 Da), generating a fragment ion at *m*/*z* 227.0712 (Δ = 0.74 ppm), corresponding to deprotonated resveratrol [M – H]^−^.

### 2.8. Monoamine Oxidase Activity Measurement

MAO-A enzymatic activity was quantified in purified brain and liver mitochondrial preparations. Mitochondrial isolation from tissue homogenates followed established protocols from Satav and Katyare [21]. Evaluation of MAO-A activity in both brain and liver tissues was conducted using methodologies adapted from Tipton et al. [22].

For MAO-A assessment, cerebral tissue homogenates were incubated with 100 μL of 0.5 μM L-deprenyl (a MAO-B-specific inhibitor) for 60 min at 37 °C prior to analysis. The reaction was initiated by introduction of 5-hydroxytryptamine creatinine sulfate (4 mM) as the MAO-A-specific substrate. Spectrophotometric quantification at 278 nm determined enzymatic activity, expressed as nanomoles of serotonin catabolized (via MAO-A pathway) per milligram protein per minute.

**Figure 2:**
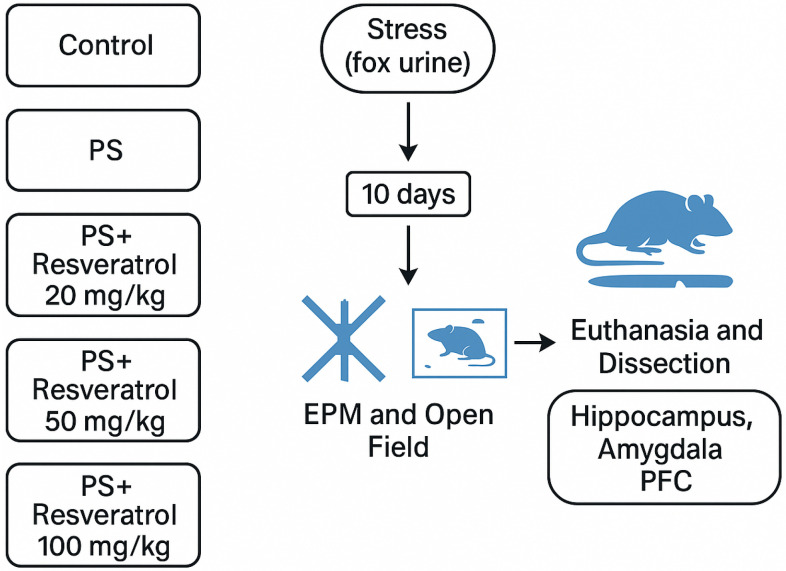
Graphical representation of the experimental workflow.

A graphical overview of the experimental design, including group allocation (control and treatment groups) and the sequence of behavioral and biochemical assessments, is presented in Figure 2.

### 2.9. Statistical Analysis

Data were analyzed using SPSS 24.0 (SPSS Inc., Chicago, IL, USA), STATISTICA 10.0 (StatSoft Inc., Tulsa, OK, USA), and MS Excel 2010 (Microsoft Inc., Redmond, WA, USA) software. Quantitative data are expressed as the mean ± standard deviation (SD). Comparisons among groups were performed using the Kruskal–Wallis test, followed by Dunn’s post hoc tests for pairwise comparisons. Spearman’s rank correlation coefficient was used to assess the relationships between variables.

The effect size was estimated based on preliminary data obtained in previous studies conducted in our laboratory, in accordance with established recommendations and relevant literature from comparable research [23].

### 2.10. Molecular Docking Protocol

The high-resolution three-dimensional (3D) structure of monoamine oxidase A (MAO-A) was retrieved from the RCSB Protein Data Bank [24]. For molecular docking studies, the crystal structure of MAO-A (PDB ID: 2Z5X) [25] was selected. In this structure, the active site of MAO-A is occupied by the co-crystallized ligand HRM (7-methoxy-1-methyl-9*H*-β-carboline). Only the A chain of the MAO-A protein was retained for docking, while all water molecules and small ligands, except for the bound prosthetic group flavin adenine dinucleotide (FAD), were removed. Protein preparation was performed using Chimera 1.14 [26], which included the assignment of Gasteiger charges to each atom and the merging of all non-polar hydrogen atoms. Atom types were assigned according to the AutoDock force field, and the processed receptor structures were saved in pdbqt format for subsequent docking simulations.

The 3D structures of resveratrol (RES), *trans*-resveratrol-3-*O*-glucuronide (ROG), and serotonin (5-HT) were retrieved from the PubChem database [27]. Ligand preparation was carried out using the Python script mk_prepare_ligand.py version 0.6.1, developed by the Forli lab at the Center for Computational Structural Biology (CCSB) [28]. This script was employed to assign Gasteiger charges, define atom types according to the AutoDock force field, and generate the required pdbqt files for molecular docking simulations.

The center of the docking grid for MAO-A was defined at coordinates (40.6, 26.9, −14.5) Å, while the grid box dimensions were set to 75 × 60 × 55 Å^3^ to encompass the entire binding site and allow sufficient conformational flexibility for ligand binding. Molecular docking simulations were conducted using AutoDock Vina version 1.2.5 [29], with an exhaustiveness parameter of 600 and a total of 100 docking modes per ligand to ensure a thorough exploration of binding conformations. Docked conformations were retained only if their binding affinity was within 4 kcal mol^−1^ of the highest-ranked pose, ensuring the selection of energetically favorable binding modes.

To assess different binding scenarios, docking simulations were performed for three sets of multiple ligands (5-HT, RES, ROG; 5-HT, RES; 5-HT, ROG) and a single-ligand (5-HT) system. Following docking, all generated poses were visually inspected, and the conformations with the lowest binding energy were selected for subsequent structural and energetic analyses.

### 2.11. Molecular Dynamics Protocol

The molecular dynamics (MD) simulations were conducted using the Amber 22 package [30].

Prior to the simulations, the protonation states of protein side chains were determined using the PDB2PQR web server [31], ensuring accurate assignment based on physiological pH conditions. Ligand parameterization was performed with the Antechamber module of Amber 22 [32], employing the General Amber Force Field 2 (GAFF2) [33] for atom type assignment. Partial atomic charges were derived using the restrained electrostatic potential (REsP) fitting method, a widely used approach that optimally reproduces the electrostatic potential around molecules. The protein was parametrized using the Amber ff19SB force field [34], which provides improved accuracy for protein–ligand interactions. To reduce computational complexity, the hydrophobic *N*-terminal helix (Val498–Leu524), which anchors MAO-A to the mitochondrial membrane was removed.

The protein–ligand complexes were solvated in an octahedral water box, maintaining a minimum distance of 12 Å between the solute and the box boundary. The OPC water model was employed due to its superior compatibility with the ff19SB force field [35]. The systems were neutralized by adding three Na+ counterions and subsequently adjusted to a physiological salt concentration (0.15 M NaCl) following the protocol by Machado and Pantano [36].

Four independent MD simulations were performed for the following systems: MAO-A complexed with 5-HT, RES, and ROG (MAO-A:5-HT:RES:ROG), MAO-A with 5-HT and RES (MAO-A:5-HT:RES), MAO-A with 5-HT and ROG (MAO-A:5-HT:ROG), and MAO-A bound to 5-HT alone (MAO-A:5-HT).

Each system underwent energy minimization using periodic boundary conditions, with harmonic restraints (*k* = 10.0 kcal mol^−1^ Å^−2^) applied to the protein, FAD, and ligands. A total of 10,000 minimization steps were performed, consisting of 4000 steps using the steepest descent algorithm, followed by 6000 steps using the conjugate gradient method.

Following minimization, the systems were gradually heated from 0 K to 310 K over 500 ps without positional restraints, ensuring a smooth transition to physiological temperature. The heating phase was followed by a 500 ps equilibration phase to allow stabilization of temperature and pressure.

A 300 ns production MD simulation was performed for each system under constant pressure (1 atm) and temperature (310 K), maintained using a Langevin thermostat with a collision frequency of 1 ps^−1^. The time step for numerical integration was set to 2 fs. Bond lengths involving hydrogen atoms were constrained using the SHAKE algorithm [37], allowing for a stable 2 fs time step. Non-bonded interactions were computed using an 11 Å cutoff, while long-range electrostatic interactions were handled using the Particle Mesh Ewald (PME) method [38]. Periodic boundary conditions were applied in all directions.

To enhance statistical reliability, all simulations were conducted in triplicate, yielding a cumulative simulation time of 900 ns. MD simulations were executed on the Supek supercomputer at the University Computing Center (SRCE), University of Zagreb, Croatia.

### 2.12. Free Energy of Binding Calculation

The binding free energy ($\Delta G_{bind}$) between the protein and 5-HT was calculated using the molecular mechanics/generalized Born surface area (MM/GBSA) method, implemented via the MMPBSA.py script from the AmberTools package [39]. The calculation followed a single-trajectory approach with the following formula:

(1) $$\Delta G_{bind}=\Delta H-T\Delta S$$

(2) $$\Delta E_{MM}=\Delta E_{inter}+\Delta E_{ele}+\Delta E_{vdW}$$

(3) $$\Delta G_{sol}=\Delta G_{GB}+\Delta G_{SA}$$

In this formulas, $\Delta E_{MM}$ represents the change in molecular mechanics energy, which includes bond, angle, and dihedral contributions ($\Delta E_{inter}$), along with electrostatic ($\Delta E_{ele}$) and van der Waals ($\Delta E_{vdW}$) energies. The solvation free energy change, $\Delta G_{sol}$, is composed of two parts: the polar ($\Delta G_{GB}$, electrostatic solvation energy) and the non-polar ($\Delta G_{SA}$, non-electrostatic solvation energy) components. Finally, TΔS accounts for the entropic contribution to binding.

During the production phase, the trajectory was divided into six segments, each spanning 50 ns. From each segment, 100 snapshots were extracted at regular intervals to ensure comprehensive sampling of conformational space. Binding free energy ($\Delta G_{bind}$) calculations were performed for each snapshot, and the final $\Delta G_{bind}$ value was reported as the mean ± standard deviation across all six segments for the three independent replicates.

Additionally, the MM/PBSA binding free energy was decomposed on a per-residue basis to evaluate the contribution of individual residues to the overall binding free energy. This decomposition enabled the identification of specific interactions and energetic contributions. Due to the high computational cost associated with calculating the entropy term, it was omitted from the analysis.

## 3. Results

### 3.1. In Vivo

#### 3.1.1. Levels of Resveratrol and Resveratrol Glucuronide in the Plasma of Stressed Animals Treated with Resveratrol at Doses of 20, 50, and 100 mg/kg

The concentrations of resveratrol and its metabolite, resveratrol glucuronide, are shown in (Table 1). At the investigated doses of RES, the concentration of its metabolite predominates, accounting for more than 95% of the total content of these stilbenoids.

#### 3.1.2. Effects of Resveratrol on Anxiety-like Behavior and MAO-A Activity in Stressed Rats

**Figure 3:**
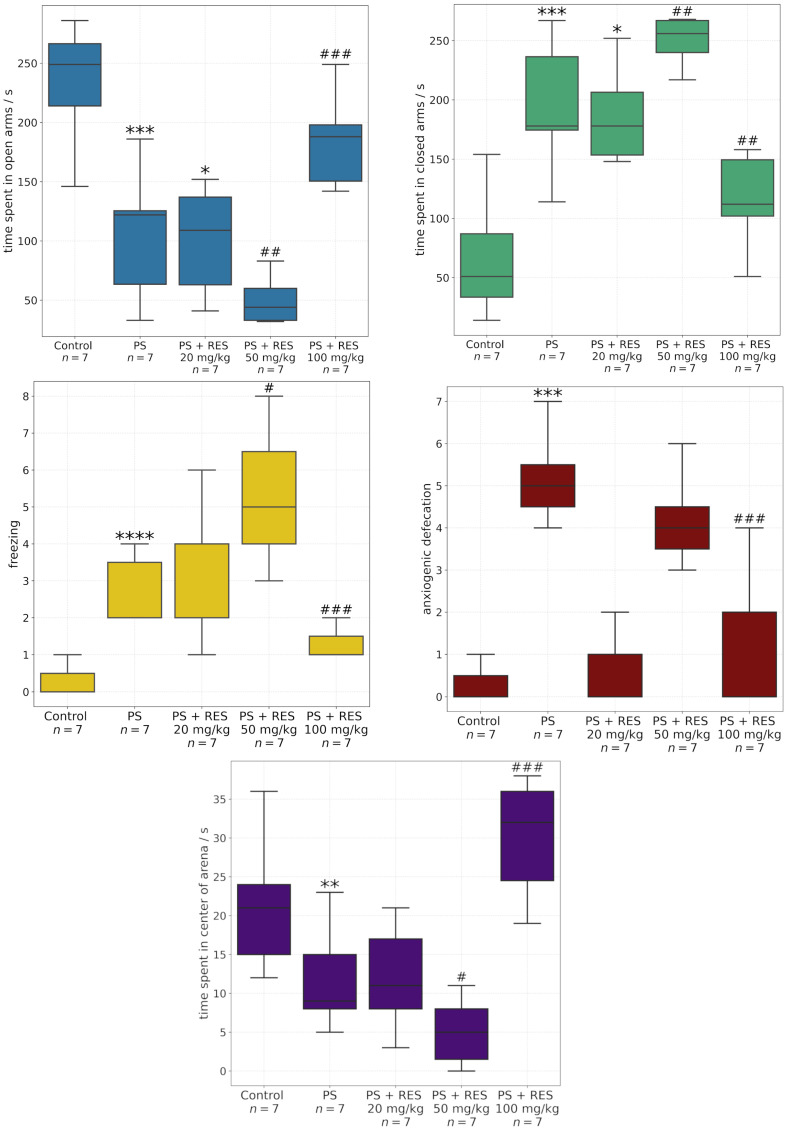
Effect of resveratrol treatment at doses of 20, 50, and 100 mg/kg on behavioral outcomes in rats subjected to predator stress. Behavioral performance was assessed using the elevated plus maze and open field tests to evaluate anxiety-like responses. * = effect between groups PS and control; ^#^ = effect between groups PS and PS + RES; * *p* < 0.01; ** *p* < 0.05; *** *p* < 0.001; **** *p* < 0.0001; ^#^ *p* < 0.05; ^##^ *p* < 0.01; ^###^ *p* < 0.001.

Behavioral outcomes in PS-exposed animals treated with RES at doses of 20 mg/kg, 50 mg/kg, and 100 mg/kg are summarized in Table 2 and Figure 3. Chronic PS induced anxiety-like behavior, as evidenced by increased freezing behavior, prolonged time spent in the closed arms, and reduced exploration of the open arms in the elevated plus maze. Additionally, chronic PS reduced the time spent in the center of the open field (OF) arena (F_4,30_ = 9.45; *p* = 0.0001), while increasing the frequency of freezing behavior (F_4,30_ = 14.13; *p* = 0.0001) and anxiety-induced defecation (F_4,30_ = 21.86; *p* = 0.0001).

RES administration at 20 mg/kg did not significantly affect the number of entries into the light and dark arms or the time spent in the open and closed arms. Resveratrol at this dose did not exert a significant effect on the behavioral parameters in the OF test. However, treatment with 50 mg/kg RES exacerbated the anxiogenic effects of chronic stress, further decreasing time spent in the open arms and increasing time in the closed arms compared to both the control and PS groups. Furthermore, resveratrol at this dose exacerbated anxiety disorders and the expression of fear, as evidenced by the OF test. In the PS + RES 50 mg/kg group, compared to the PS group, there was a reduced time spent in the center of the arena and increased levels of anxiety-induced defecation and freezing behavior. In contrast, 100 mg/kg RES alleviated stress-induced anxiety-like behavior, restoring time spent in the open and closed arms to control levels.

**Figure 4:**
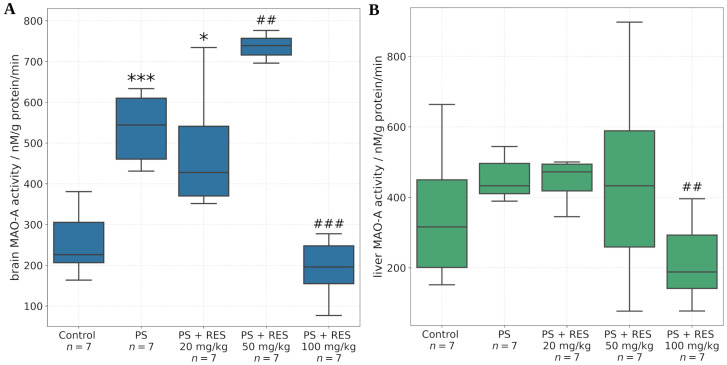
Effect of predator stress (PS) and resveratrol (RES) at doses of 20, 50, and 100 mg/kg on MAO-A activity in the brain (**A**) and liver (**B**). * = effect between groups PS and control; ^#^ = effect between groups PS and PS + RES; * *p* < 0.01; *** *p* < 0.001; ^##^ *p* < 0.01; ^###^ *p* < 0.001.

Figure 4 illustrates the effects of different RES doses on MAO-A activity in the brain and liver. In stressed animals, a significant increase in MAO-A activity was observed in the brain. RES treatment at 20 mg/kg failed to prevent stress-induced elevation of MAO-A activity. Notably, administration of 50 mg/kg RES resulted in a twofold increase in brain MAO-A activity compared to control levels (F_2,36_ = 4.21; *p* = 0.022). In contrast, 100 mg/kg RES completely mitigated the stress-induced rise in brain MAO-A activity.

Unlike the brain, the liver of stressed animals did not exhibit increased MAO-A activity. However, administration of 100 mg/kg RES led to a twofold reduction in hepatic MAO-A activity. Lower doses of RES (20 mg/kg and 50 mg/kg) did not produce significant changes in hepatic MAO-A activity.

**Figure 5:**
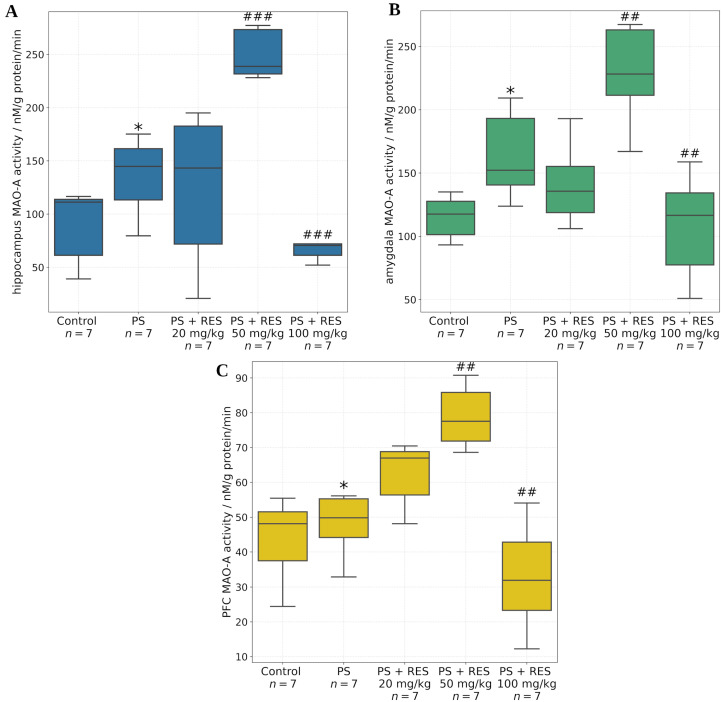
Effect of predator stress (PS) and resveratrol (RES) at doses of 20, 50, and 100 mg/kg on MAO-A activity in the hippocampus (**A**), amygdala (**B**), and prefrontal cortex (PFC, (**C**)). * = effect between groups PS and control; ^#^ = effect between groups PS and PS + RES; * *p* < 0.05; ^##^ *p* < 0.05; ^###^ *p* < 0.001.

Figure 5 illustrates changes in MAO-A activity in the amygdala, hippocampus, and prefrontal cortex. It was found that PS was associated with increased MAO-A activity in both the hippocampus and amygdala. Resveratrol at a dose of 100 mg/kg reduced MAO-A activity, whereas resveratrol at a dose of 50 mg/kg enhanced MAO-A activity in all brain regions examined. Resveratrol at a dose of 20 mg/kg had no effect on MAO-A activity in these brain regions.

### 3.2. In Silico

Molecular docking revealed that 5-HT binds to the active site of the MAO-A enzyme in all simulated complexes, located in close proximity to the prosthetic group FAD Figure S1. In the MAO-A:5-HT:ROG complex, the ligand ROG adopts a position almost parallel to α-helix H3 and is situated near helix H24 (for nomenclature see Figure S2) at the *C*-terminal region of the enzyme. A similar binding position is observed for the ligand RES in the MAO-A:5-HT:RES complex, where it also binds at the same site between H3 and H24. In contrast, in the complex with three ligands (MAO-A:5-HT:RES:ROG), RES binds to the surface of the enzyme near residue Asp36, while ROG remains bound within the same pocket as in the two-ligand complex. The position of 5-HT is highly conserved across all complexes, with only minor differences in the relative orientation of its NH2 group. These docked geometries were subsequently used as input structures for molecular dynamics (MD) simulations.

To investigate the influence of RES and its derivative, ROG, on the binding of 5-HT to MAO-A, we performed four sets of molecular dynamics (MD) simulations. These included the MAO-A complex with serotonin alone (MAO-A:5-HT), as well as systems where serotonin was co-bound with resveratrol (MAO-A:5-HT:RES), its derivative (MAO-A:5-HT:ROG), or both compounds simultaneously (MAO-A:5-HT:RES:ROG).

Each system was subjected to 300 ns of MD simulations, conducted in triplicate, to ensure statistical robustness. Our primary objective was to assess how the presence of RES and/or ROG modulates the stability and binding interactions of 5-HT within the MAO-A active site. To this end, we analyzed the structural dynamics, binding free energies, and key intermolecular interactions in each complex, providing insights into potential allosteric effects or competition among the ligands.

**Figure 6:**
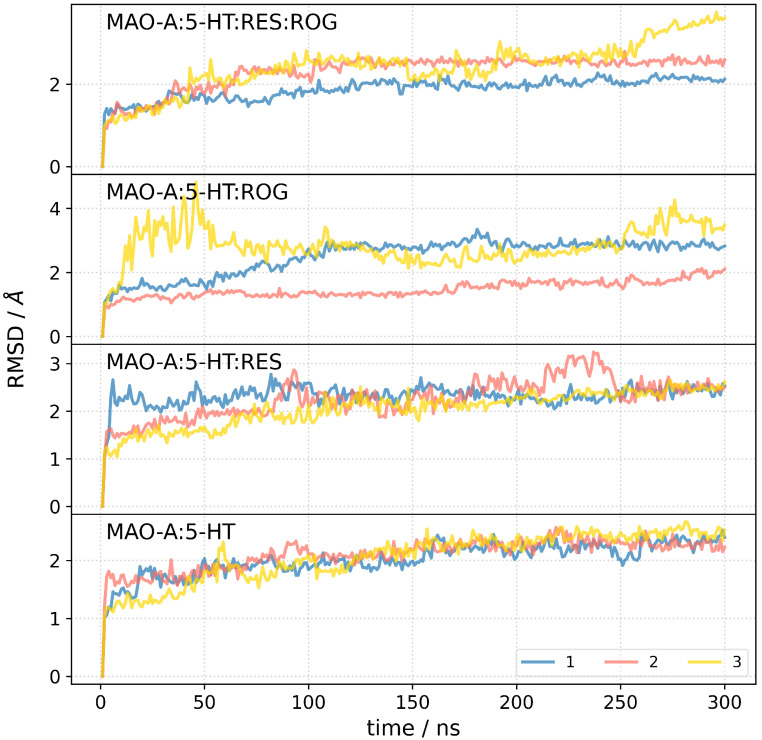
Root mean square deviation (RMSD) profiles of MAO-A complexes over 300 ns molecular dynamics simulations in triplicate.

To assess the structural stability of the MAO-A:ligand complexes, we monitored the root mean square deviation (RMSD) of the protein backbone over 300 ns for all four systems (Figure 6). The RMSD profiles indicate that all complexes reached equilibrium within the first 50 ns, after which they exhibited stable fluctuations. The MAO-A:5-HT complex displayed the lowest RMSD values, suggesting minimal conformational rearrangements upon serotonin binding. The addition of RES resulted in slightly increased RMSD values, indicating moderate structural adaptation. The presence of ROG, either alone or in combination with RES, led to higher RMSD fluctuations, particularly in one replicate of the MAO-A:5-HT:ROG complex, where RMSD exhibited a sudden increase after 50 ns and fluctuated around 4 Å. This suggests that ROG binding induces greater conformational flexibility in MAO-A, potentially altering its binding pocket dynamics. Notably, the MAO-A:5-HT:RES:ROG complex exhibited a relatively stable RMSD profile compared to MAO-A:5-HT:ROG, implying that RES may mitigate the structural perturbations introduced by ROG.

The structural compactness and overall stability of the MAO-A complexes were assessed through the analysis of the radius of gyration (R_*g*_, Figure S3) and solvent-accessible surface area (SASA) over the 300 ns MD simulations, performed in triplicate. R_*g*_ values remained relatively stable throughout the simulations, indicating that no major conformational changes or unfolding events occurred in the protein-ligand complex during the simulation. The low standard deviations further support the consistency of the protein’s compact structure across the different runs (Table 3). SASA refers to the surface area of a biomolecule that is accessible to a solvent (water in our case). It is a crucial metric in understanding the exposure of residues or ligands to the solvent environment and is often used in MD simulations to monitor structural changes over time. The mean SASA values show that the protein’s surface exposure to the solvent also remained relatively unchanged. The small variations in SASA suggest that no large-scale structural changes took place, which is in agreement with the R_*g*_ data.

The root mean square fluctuation (RMSF) analysis was conducted to investigate residue-level flexibility in MAO-A across all four complexes Figure S4. The RMSF profiles demonstrate consistent trends across the replicates, underscoring the reproducibility of the simulations. As shown in the plots, residues located at the *N*- and *C*-terminal regions exhibit higher RMSF values, indicative of increased flexibility in these unstructured regions. In contrast, residues within the core structured regions of the protein, particularly those forming secondary structure elements such as α-helices and β-sheets, display lower RMSF values, signifying their relative rigidity. Prior to the simulation, a *N*-terminal helix (Val498–Leu524), which is typically embedded in the mitochondrial membrane, was removed to reduce the system size. This helix is known to play a role in stabilizing the protein’s position in the membrane environment. Its removal may have led to increased flexibility and movement of the nearby unstructured regions, particularly the loop, resulting in a conformational shift and the corresponding jump in RMSD. However, these structural changes are located far from the active site and binding pocket, suggesting that they are unlikely to have a significant impact on the binding energy estimation or the stability of the ligand within the binding pocket. Interestingly, residues in proximity to the active site exhibit minimal fluctuations, suggesting that this region remains structurally stable during the simulations. This stability is critical for maintaining the enzymatic function of MAO-A and ensuring proper ligand binding.

Visual inspection of the simulation trajectories supports the RMSF analysis, revealing that the most significant structural changes occur at the *C*-terminus, which exhibits high flexibility across all complexes. In the MAO-A:5-HT:ROG complex, ROG is bound between α-helix H3 and the unstructured loop preceding helix H24 (Figure S2). This loop undergoes a conformational shift, moving away from the protein surface and contributing to increased RMSD and R_*g*_ values. Similarly, in the MAO-A:5-HT:RES complex, RES occupies the same binding pocket as ROG, and this loop also displays notable flexibility. In contrast, in the MAO-A:5-HT complex without ROG or RES, this loop remains the most flexible region, suggesting that its dynamic nature may be intrinsic to its structural role. Interestingly, in complexes without ROG or RES, the *C*-terminus becomes the most flexible region, as indicated by elevated RMSF values. Across all systems, residues spanning Val481 to Ser497 consistently show high flexibility, highlighting their dynamic behavior. These findings suggest that while ligand binding induces localized structural changes, particularly in loop regions near the binding pocket, the *C*-terminus may possess intrinsic flexibility independent of ligand presence. In addition to these measures, secondary structure conservation was analyzed using the DSSP algorithm [40], which assigns secondary structure elements based on hydrogen bond patterns and backbone geometries. The analysis (Figure S5) confirmed that the protein’s secondary structure remained conserved across all triplicates, further supporting the conclusions drawn from the R_*g*_ and SASA data.

These results suggest that serotonin binding alone does not significantly perturb MAO-A stability, whereas the presence of ROG induces structural fluctuations, potentially affecting ligand binding and enzymatic function.

The analysis of hydrogen bonds between 5-HT and MAO-A reveals a moderate interaction strength throughout the MD simulations. The MAO-A:5-HT complex maintains a mean of 1.9 hydrogen bonds during interactions, higher than complexes with allosteric ligands (1.3–1.8). The free energy decomposition analysis (Table 4) identified FAD (flavin adenine dinucleotide), aromatic residues (Phe208, Phe352, Tyr408), aliphatic residues (Ile180, Leu337), and polar residues (Gln215, Asn181) as the primary contributors to ligand binding in MAO-A. The flavin cofactor (FAD) exhibited the strongest contribution, attributed to electrostatic interactions critical for substrate oxidation. Aromatic residues (Phe208, Phe352, Tyr408) dominated via π–π stacking and hydrophobic packing, stabilizing the ligand within the hydrophobic core of the binding pocket. Aliphatic residues (Ile180, Leu337) enhanced binding through van der Waals interactions, optimizing shape complementarity. This synergy between hydrophobic stabilization and localized polar interactions underscores MAO-A’s reliance on aromatic-rich motifs for substrate recognition, with FAD’s electrostatic role aligning with its catalytic function in neurotransmitter metabolism.

The MM/GBSA binding free energy analysis for the MAO-A:5-HT complex (Table 5), both in the presence and absence of resveratrol and its metabolite *trans*-resveratrol-3-*O*-glucuronide, revealed key energetic contributions governing ligand binding. The total binding free energy ($\Delta G_{bind}$) for the MAO-A:5-HT complex was calculated as −17.9 ± 2.6 kcal mol^−1^, with van der Waals interactions ($\Delta E_{vdW}$) being the dominant stabilizing factor (−24.7 ± 1.8 kcal mol^−1^), followed by electrostatic interactions ($\Delta E_{ele}$, −20.7 ± 7.4 kcal mol^−1^). The solvation free energy ($\Delta G_{GB}+\Delta G_{SA}$) contributed unfavorably, particularly the polar desolvation energy ($\Delta G_{GB}$), which counteracted favorable electrostatic contributions. The presence of RES and ROG slightly modulated these interactions, with minor variations in binding energy, suggesting that their inclusion does not drastically alter the fundamental binding mode of 5-HT. However, the slight decrease in $\Delta G_{bind}$ upon adding ROG (−15.5 ± 2.3 kcal mol^−1^) suggests a minor destabilization effect.

These findings align well with the binding free energy decomposition analysis, which identified key residues contributing to ligand stabilization. The dominance of van der Waals interactions is consistent with the significant roles of hydrophobic residues such as Phe208, Phe352, Ile180, Leu337, and Tyr408, which facilitate ligand binding via π–π stacking and nonpolar stabilization. Meanwhile, the electrostatic contributions reflect the involvement of polar residues such as Gln215 and Asn181. The unfavorable desolvation energy further emphasizes the importance of the hydrophobic environment within the binding pocket, which protects 5-HT from solvent exposure. The relatively stable binding energy across conditions suggests that the core interactions between 5-HT and MAO-A remain largely conserved, reinforcing the critical role of identified residues in defining ligand affinity. These insights provide a detailed energetic framework for understanding serotonin binding and could guide future modifications aimed at optimizing interactions within the MAO-A active site.

## 4. Discussion

This article is part of a comprehensive study demonstrating the therapeutic effects of RES. In earlier works, we have shown the protective effects of RES in experimental PTSD and under conditions of chronic stress, which serve as a trigger in this PTSD model. Overall, it has been established that RES reduces anxiety-like behavior by modulating the regulation of key enzymes such as 11β-hydroxysteroid dehydrogenase type 1 (11β-HSD-1) and monoamine oxidase. Thus, RES exerts anti-anxiogenic effects by modulating the metabolism of glucocorticoids and monoamines in both the brain and liver [18].

Furthermore, under conditions of predator stress and PTSD, the therapeutic effects of RES were manifested in hepatoprotective activity, evidenced by a reduction in necrotic liver damage and inflammation in the organ. It was also found that chronic exposure to predator stress led to a significant increase in serotonin levels and upregulation of the expression of the SERT and 5-HT_3*A*_ receptors. SSRIs were unable to prevent anxiety or reduce serotonin levels, partly due to suppressed SERT expression. RES reduced the regulation of SERT and 5-HT_3*A*_ expression to a lesser extent than SSRIs, but effectively decreased anxiety and restored serotonin levels, likely through upregulation of MAO-A expression. Furthermore, this study compared the therapeutic effects of RES with those of SSRIs. It was found that none of the four analyzed drugs were able to effectively influence behavioral disorders under stress conditions. Therefore, the therapeutic effect of RES was superior to that of the other drugs. However, in these studies, a bidirectional effect of RES at a dose of 100 mg/kg on gene expression and MAO-A activity was identified [12].

Despite the observed increase in MAO-A gene expression, administration of RES in stressed animals resulted in a paradoxical reduction in MAO-A activity, which correlated with an attenuation of stress-induced anxiety-like behavior. Under conditions of increased MAO-A production, a substantial enhancement of the enzyme’s activity is likely. Consistent with this hypothesis, our previous studies demonstrated that RES upregulates MAO-A gene expression while downregulating serotonin transporter (SERT) gene expression in the hippocampus of chronically stressed animals [12,41]. Through increased MAO-A expression and activity, RES may exert pro-anxiogenic effects, particularly given that serotonin is a primary substrate of MAO-A. Notably, in our experimental model, MAO-A activity was measured based on the oxidative deamination of serotonin, and a reduction in serotonin levels is well-documented to contribute to stress-related anxiety and depressive disorders [42].

Therefore, in the present study, we intentionally narrowed the focus to a comparison of in silico predictions with in vivo findings regarding the modulation of MAO-A activity. Anxiety-related behaviors were evaluated using a range of behavioral paradigms, including the elevated plus maze and the open field test. Predatory stress was associated with a marked decrease in the time spent in the open arms of the EPM and the center of the OF arena. Additionally, a significant increase in spontaneous freezing behavior—a validated index of fear response—was observed in the OF test.

RES exerted a dose-dependent effect on behavioral markers of anxiety and fear. At a dose of 100 mg/kg, RES demonstrated robust anxiolytic properties and concurrently reduced fear-related responses. In contrast, at a dose of 50 mg/kg, RES elicited pro-anxiogenic effects in both the EPM and OF paradigms. Administration of resveratrol at 20 mg/kg had no statistically significant impact on behavioral activity.

The in vivo findings further demonstrated that RES modulates MAO-A activity in a dose-dependent manner across several tissues and brain regions, including the liver, whole brain, amygdala, hippocampus, and prefrontal cortex. The amygdala is a central brain structure involved in the generation of anxiety-related behaviors, and its activity is regulated through its neuronal interactions with the prefrontal cortex and hippocampus.

It was found that PS significantly increased MAO-A activity in the amygdala and hippocampus, but not in the prefrontal cortex. Positive correlations were identified between MAO-A activity in the amygdala and the time spent in the closed arms of the EPM (r = 0.72; *p* < 0.05), MAO-A activity in the hippocampus and the freezing response (r = 0.69; *p* < 0.05), and MAO-A activity in the liver and the level of anxiety-related defecation (r = 0.79; *p* < 0.05).

RES exerted dose-dependent corrective effects on MAO-A activity in the aforementioned brain regions. A significant reduction in MAO-A activity was observed in the amygdala, hippocampus, prefrontal cortex, and whole brain following administration of RES at 100 mg/kg. Conversely, MAO-A activity was increased at a dose of 50 mg/kg. No significant effect was observed with the 20 mg/kg dose in any of the brain regions examined.

In the present study, we extended our analysis by assessing hepatic MAO-A activity in addition to its enzymatic activity in the brain. A novel correlation was identified between hepatic MAO-A activity and the time spent in the dark arms of the elevated plus maze (r = 0.85, *p* < 0.05). Furthermore, a positive correlation was found between hepatic MAO-A activity and plasma concentrations of ROG (r = 0.77, *p* < 0.05).

Based on these findings, we propose that RES and its metabolites interact with an allosteric site on MAO-A, leading to enzyme inhibition. This hypothesis is supported by our in silico analyses, which provide further evidence for resveratrol-mediated allosteric modulation of MAO-A activity.

Statistical analysis using a *t*-test revealed that the calculated binding free energies ($\Delta G_{bind}$) for the different complexes were not significantly different, indicating that the presence of resveratrol (RES) and its metabolite trans-resveratrol-3-O-glucuronide (ROG) does not substantially alter the thermodynamic stability of serotonin (5-HT) binding to MAO-A. However, experimentally observed reductions in MAO-A activity in the presence of RES and ROG suggest an additional regulatory mechanism not accounted for in the present study. Given that our analysis focused on binding free energy calculations, we explored the potential for allosteric modulation by RES and ROG rather than direct competitive inhibition. Nevertheless, we did not explicitly investigate the influence of these compounds as competitive inhibitors, leaving open the possibility that their impact on enzyme activity could arise from alternative mechanisms such as conformational changes or indirect effects on enzyme dynamics. Further studies integrating kinetic assays and enhanced sampling molecular dynamics simulations would be required to fully elucidate these effects.

Notably, in addition to RES, its glucuronide metabolite exhibits a distinct affinity for the allosteric site of MAO-A. Our previous studies demonstrated significant correlations between behavioral activity measures and plasma concentrations of ROG in stressed animals. Specifically, a negative correlation was observed between time spent in the open arms of the elevated plus maze and plasma levels of ROG.

The potential allosteric effects of RES/ROG identified in silico may represent integral components of the molecular and systemic mechanisms underlying the anxiolytic effects of RES. The molecular mechanisms of RES’s action are primarily associated with its ability to function as a ligand for sirtuins—deacetylase enzymes. In various types of neuronal cultures, the protective effects of RES against mitochondrial dysfunction, oxidative stress, and apoptosis have been shown to be enhanced via upregulation of SIRT1 expression [43]. SIRT1, in turn, activates multiple molecular pathways and mediates a range of neuroprotective effects of RES treatment.

These neuroprotective effects are closely linked to the improvement of mitochondrial function. RES enhances mitochondrial efficiency through activation of the SIRT1/AMPK/PGC-1α signaling axis. Additionally, RES promotes neuroplasticity via the SIRT1/AMPK/CREB/BDNF pathway, thereby supporting synaptic plasticity and overall neurotransmission. By downregulating NF-κB, RES reduces the release of pro-inflammatory cytokines from glial cells, alleviating neuroinflammation and oxidative stress—processes that are critically involved in the pathophysiology of stress-related anxiety disorders. The restoration of neurotransmission under such conditions constitutes a key neuroprotective mechanism of RES.

The systemic mechanisms underlying the anxiolytic effects of RES have been comprehensively reviewed [44]. This analysis convincingly demonstrated that key components of the pathogenesis of stress-related anxiety disorders—including neuroinflammation, oxidative stress, mitochondrial dysfunction, impaired neuroplasticity, dysregulated neuronal circuitry and neurotransmitter levels, altered cerebral blood flow, dysfunction of the liver–hypothalamic–pituitary–adrenal (LHPA) axis, and disturbances in the gut–brain and liver–brain axes—are amenable to modulation by RES [44].

The therapeutic actions of RES are primarily based on its capacity to counteract neuroinflammation, oxidative stress, and mitochondrial dysfunction. As a potent antioxidant, RES may exert its protective effects via direct scavenging of reactive oxygen species. Oxidative stress is a major contributor to mitochondrial dysfunction, and it is noteworthy that MAO-A is localized on the outer mitochondrial membrane. This spatial proximity allows for the direct mitochondrial penetration of RES and its potential interaction with allosteric sites on MAO-A.

RES has also been shown to enhance synaptic structure and function by increasing dendritic spine density and upregulating the expression of postsynaptic density protein 95 (PSD95) and brain-derived neurotrophic factor (BDNF), thereby mitigating paclitaxel-induced synaptic damage [45]. Beyond BDNF and glial-cell-line-derived neurotrophic factor (GDNF), RES activates signaling pathways such as ERK1/2 and CREB [46]. In vitro, the neuroprotective effects of RES are associated with increased levels of SIRT1, phosphorylated CREB (p-CREB), total CREB, and BDNF, as well as reduced expression of miR-134 [46]. Moreover, RES enhances the expression of synaptic plasticity-related proteins, including SynGAP, PSD95, synapsin-1, and synaptotagmin-1 in the hippocampus, in a SIRT1-dependent manner [45,47].

The ability of RES to improve synaptic plasticity is particularly relevant for addressing stress-related anxiety disorders, where synaptic stability is critical for efficient neurotransmitter function. Collectively, these neuroprotective properties contribute to the therapeutic potential of RES in alleviating anxiety symptoms through enhanced synaptic resilience and improved neurotransmission.

The localization of MAO-A on the outer mitochondrial membrane is a crucial factor influencing its activity, as the enzyme is highly sensitive to the state of its lipid microenvironment [22]. Previous in vivo and in vitro studies have demonstrated that oxidative stress-induced lipid peroxidation in mitochondrial membranes can lead to MAO-A inactivation [22]. Given resveratrol’s lipophilic nature, it readily penetrates mitochondria and acts as a free radical scavenger, exerting potent antioxidant effects [48]. In this context, RES likely serves a protective role not only for MAO-A but also for other mitochondrial membrane proteins.

However, MAO-A is an oxidase that generates H2O2 as a byproduct, which, in the presence of Fe2+ and Cu2+ ions, can contribute to oxidative stress. RES may provide additional protection against the auto-oxidation of MAO-A [49]. This raises the possibility that the observed upregulation of MAO-A activity in response to RES is mediated through multiple mechanisms. One such mechanism could involve the SIRT1/NHLH2/MAO-A pathway, in which SIRT1-induced deacetylation of NHLH2 transcription factors leads to increased MAO-A expression and decreased 5-HT levels in neurons, potentially exacerbating anxiety-like behaviors [44,50].

It is plausible that the anxiogenic effects of RES at a dose of 50 mg/kg are mediated through this mechanism. Notably, at this dose, RES increased MAO-A activity in the amygdala, hippocampus, and prefrontal cortex, as well as in whole-brain homogenates, but not in the liver. Glucocorticoids are known to enhance MAO-A gene expression in multiple brain regions [51]. Therefore, RES at 50 mg/kg may potentiate both the SIRT1/NHLH2/MAO-A signaling axis and glucocorticoid synthesis [52]. In turn, elevated glucocorticoid levels further upregulate MAO-A expression.

Importantly, administration of RES at 50 mg/kg also increased spontaneous freezing behavior in the OF test, a response indicative of heightened fear. Such fear responses are known to be potentiated by glucocorticoid dysregulation [53].

In contrast, at a dose of 100 mg/kg, the putative allosteric effects of RES may be engaged, as reflected by the generalized suppression of MAO-A enzymatic activity across all investigated brain regions and the liver.

**Figure 7:**
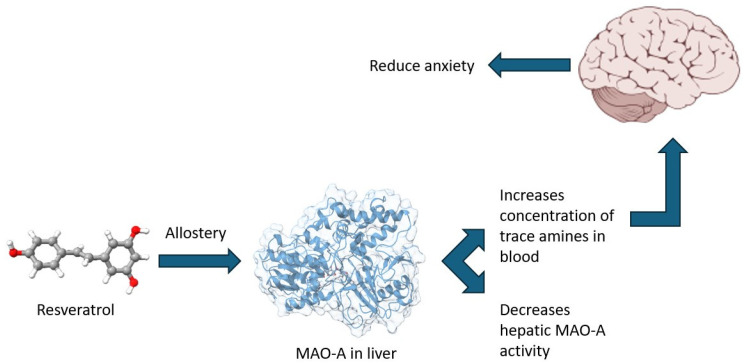
Proposed mechanism of hepatic MAO-A contribution to reduced anxiety.

Mechanistically, the association between brain MAO-A activity/expression and anxiety-related behaviors is well established due to its role in neurotransmitter metabolism. However, the involvement of hepatic MAO-A in anxiety regulation is less apparent. Nevertheless, it is important to highlight the ability of hepatic MAO-A to catalyze the oxidative deamination of monoamines of intestinal origin, which are produced through the decarboxylation of amino acids by the gut microbiota [54]. Thus, it can be hypothesized that hepatic MAO-A plays a key role in regulating the gut–liver–brain axis by modulating the levels of trace amines such as tyramine, tryptamine, phenylethylamine, and others. These amines enter the liver via the portal vein, where they are partially metabolized by hepatic MAO-A before reaching the systemic circulation and subsequently crossing the blood–brain barrier (Figure 7). In the brain, trace amines interact with their specific receptor system-trace amine-associated receptors (TAAR), which comprise nine known subtypes. Notably, TAAR1 colocalizes with dopamine D2 receptors, and its activation has been implicated in anti-addictive, antipsychotic, anxiolytic, and antidepressant effects [55]. Moreover, recent studies have demonstrated that TAAR1 agonists can ameliorate experimental PTSD [56].

Given the strong positive correlation between hepatic MAO-A activity and anxiety-related behaviors (r = 0.85, *p* < 0.05), we propose the existence of a distinct mechanism underlying anxiety development during chronic stress. Hepatic MAO-A, through oxidative deamination, may reduce the availability of trace amines necessary for brain function under chronic stress conditions. Simultaneously, brain MAO-A dysregulates neurotransmitter metabolism, further exacerbating anxiety-like behaviors.

The anxiolytic effects of RES appear to be mediated not only through inhibition of brain MAO-A but also via suppression of hepatic MAO-A activity. Interestingly, RES significantly inhibited both hepatic and brain MAO-A activity only at the highest dose (100 mg/kg), whereas no statistically significant differences were observed between stressed (PS) and resveratrol-treated (PS + RES) groups at lower doses. In silico analyses provide valuable insights into this phenomenon, suggesting that the allosteric binding sites of MAO-A may exhibit sensitivity to high concentrations of RES. This hypothesis may also extend to ROG.

Based on the comparison of in vivo data with in silico findings, a hypothesis emerged suggesting that the differences in cellular conditions (such as pH and ion composition) between the liver and brain, or the varying concentrations of RES and its metabolite, ROG, in these tissues, could explain the observed differences in their sensitivity to resveratrol and its metabolite. This is supported by the distinct correlation patterns observed: RES was correlated exclusively with brain MAO-A, while ROG was associated only with hepatic MAO-A. Future studies will further investigate this hypothesis and explore the broader biological effects of resveratrol glucuronide.

Currently, most research focuses on the protective effects of RES, while its metabolites remain relatively understudied. Nevertheless, emerging evidence highlights the therapeutic potential of ROG. Notably, preconditioning neuronal cultures with low concentrations (0.01–10 nM) of ROG protected neurons from serum withdrawal-induced apoptosis via cAMP-mediated signaling pathways [57]. It is plausible that the neuroprotective effects of ROG underlie its ability to mitigate stress-related behavioral alterations. Furthermore, the systemic effects of RES and its metabolite may be integrated within the gut–liver–brain axis, emphasizing the need for further research in this area.

The therapeutic mechanisms of RES presented herein have been characterized primarily in male subjects. Whether resveratrol elicits comparable effects in females remains an open question. This issue is of particular importance given that post-traumatic stress disorder (PTSD) and anxiety disorders often present with greater severity in females compared to males. Moreover, estrogens are known to regulate MAO-A activity [58], suggesting potential sex-specific differences in the neurobiological response to RES.

## 5. Conclusions

In this study, we provided an in silico rationale for the potential binding of resveratrol and its metabolite, resveratrol glucuronide, to the allosteric site of MAO-A. In experimental studies conducted on a chronic predator stress model, we demonstrated that the anti-anxiogenic effects of resveratrol are associated with the inhibition of not only the brain but also the liver isoform of MAO-A activity. Thus, we uncovered new insights into the involvement of the liver–brain axis. Further research will explore the relationship between liver MAO activity levels and the concentrations of trace amines in the blood, liver, and brain. Additionally, in silico investigations will be conducted to determine the binding sites of resveratrol and resveratrol glucuronide on MAO-B. This is particularly important in the context of MAO-B’s role in trace amine metabolism.

## Figures and Tables

**Table 1:** Levels of resveratrol (RES) and resveratrol glucuronide (ROG) in plasma of rats (M ± m) ^1^.

Group	RES (mkg/mL)	ROG (mkg/mL)
PS + RES 100 mg/kg	0.73 ± 0.14 (3.5%)	19.67 ± 2.2 (96.5%)
PS + RES 50 mg/kg	0.49 ± 0.11 (3.3%)	14.21 ± 1.14 (96.7%)
PS + RES 20 mg/kg	0.26 ± 0.07 (3.1%)	8.11 ± 2.3 (96.1%)

^1^ The percentage content of RES and ROG relative to their total content is given in parentheses.

**Table 2:** Effect of resveratrol treatment at doses of 20, 50, and 100 mg/kg on the behavior of rats subjected to PS.

Group	Entries in the Open Arms	Entries in the Closed Arms
Control (*n* = 7)	3.1 ± 0.21	2.45 ± 0.62
PS (*n* = 7)	1.3 ± 0.09	2.11 ± 0.41
PS + RES 20 mg/kg (*n* = 7)	1.9 ± 0.9	1.73 ± 0.97
PS + RES 50 mg/kg (*n* = 7)	1.45 ± 0.21	1.6 ± 0.34
PS + RES 100 mg/kg (*n* = 7)	2.73 ± 0.48	1.93 ± 0.61

**Table 3:** Average values and standard deviations of RMSD (in Å), radius of gyration (R_*g*_) (in Å), solvent-accessible surface area (SASA) (in Å^2^), root-mean-square fluctuation (RMSF) (in Å) and number of hydrogen bonds for the MAO-A:ligand complexes, calculated from 300 ns molecular dynamics trajectories performed in triplicate.

	RMSD	R_*g*_	SASA	RMSF	H-bond
MAO-A:5-HT:RES:ROG	2.21 ± 0.51	23.2 ± 0.1	22150 ± 446	1.30 ± 0.84	1.32 ± 1.01
MAO-A:5-HT:ROG	2.27 ± 0.75	23.2 ± 0.2	22150 ± 446	1.31 ± 0.94	1.81 ± 1.11
MAO-A:5-HT:RES	2.21 ± 0.29	23.3 ± 0.1	21962 ± 517	1.25 ± 0.81	1.39 ± 1.08
MAO-A:5-HT	2.07 ± 0.34	23.2 ± 0.1	21842 ± 521	1.31 ± 0.78	1.90 ± 1.30

**Table 4:** Contributions (in kcal mol^−1^) of the most crucial amino acid residues for the binding of 5-HT to MAO-A.

MAO-A:5-HT:RES:ROG		MAO-A:5-HT:ROG		MAO-A:5-HT:RES		MAO-A:5-HT	
FAD	−1.67	Gln215	−1.74	FAD	−1.73	FAD	−1.52
Phe208	−1.09	FAD	−1.19	Gln215	−1.44	Tyr408	−1.05
Phe352	−0.92	Phe208	−1.08	Phe208	−0.91	Ile180	−1.02
Ile180	−0.89	Asn181	−1.06	Phe352	−0.89	Phe208	−1.01
Gln215	−0.88	Ile180	−0.74	Leu337	−0.79	Gln215	−0.99

**Table 5:** Energy analysis for binding of 5-HT to MAO-A as obtained by MM/GBSA method. All units are kcal mol^−1^.

	MAO-A:5-HT:RES:ROG	MAO-A:5-HT:ROG	MAO-A:5-HT:RES	MAO-A:5-HT
$\Delta E_{vdW}$	−24.9 ± 2.0	−24.3 ± 1.8	−24.1 ± 2.0	−24.7 ± 1.8
$\Delta E_{ele}$	−17.6 ± 3.3	−18.4 ± 3.7	−18.0 ± 4.3	−20.7 ± 7.4
$\Delta G_{GB}$	29.3 ± 2.9	30.7 ± 3.2	29.1 ± 3.2	31.0 ± 6.0
$\Delta G_{SA}$	−3.5 ± 0.2	−3.6 ± 0.3	−3.5 ± 0.2	−3.5 ± 0.2
$\Delta G_{bind}$	−16.7 ± 2.9	−15.5 ± 2.3	−16.6 ± 2.6	−17.9 ± 2.6

## Data Availability

The Amber trajectories of MAO-A:ligand complexes are openly available in the FULIR repository at https://urn.nsk.hr/urn:nbn:hr:241:401110 (accessed on 26 March 2025).

## Supplementary Materials

The following supporting information can be downloaded at: https://www.mdpi.com/article/10.3390/biomedicines13051196/s1, Figure S1: Geometries of docked complexes. MAO-A:5-HT:RES:ROG (A), MAO-A:5-HT:ROG (B), MAO-A:5-HT:RES (C), MAO-A:5-HT (D); Figure S2: Secondary structure of the MAO-A protein (pdb ID: 2Z5X). Figure is generated using PDBsum web server [59]; Figure S3: Radius of gyration (R_*g*_) profiles of MAO-A complexes over 300 ns molecular dynamics simulations in triplicate; Figure S4: Root mean square fluctuation (RMSF) profiles of MAO-A complexes over 300 ns molecular dynamics simulations in triplicate; Figure S5: Changes in the secondary structure of the MAO-A:ligand complexes during molecular dynamics simulations in triplicates. MAO-A:5-HT:RES:ROG (A), MAO-A:5-HT:ROG (B), MAO-A:5-HT:RES (C), MAO-A:5-HT (D).
