# Supplementary material for: Resveratrol and Its Metabolite as Potential Allosteric Regulators of Monoamine Oxidase A Activity in the Brain and Liver Under Chronic Predator Stress
Source: Biomedicines. 2025 May 14;13(5):1196. doi: 10.3390/biomedicines13051196 (PMC12109230; doi:10.3390/biomedicines13051196)
Supplement: Supplementary file 1 [file biomedicines-13-01196-s001.zip › biomedicines-3579978-supplementary.pdf]

Supplementary Materials: Resveratrol and Its Metabolite as Potential Allosteric Regulators of Monoamine Oxidase A Activity in the Brain and Liver Under Chronic Predator Stress

Jurica Novak [1,*], Olga B. Tseilikman [2], Vladislav A. Shatilov [2], Maxim S. Zhukov [2], Vadim A. Shevyrin [3], Zuhra R. Khismatullina [4], Albina M. Fedorova [4], Georgiy N. Patrikyan [2], Timur L. Khaibullin [2] and Vadim E. Tseilikman [2,4,5,*]

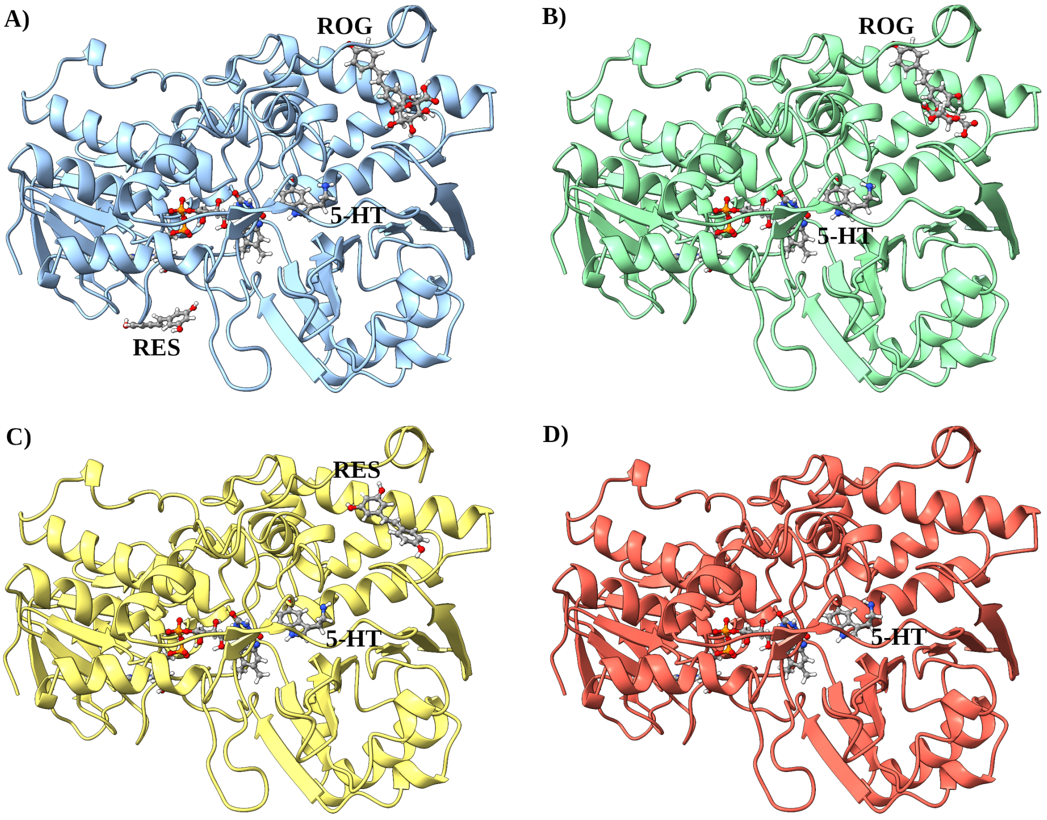

Figure S1. Geometries of docked complexes. MAO-A:5-HT:RES:ROG (**A**), MAO-A:5-HT:ROG (**B**), MAO-A:5-HT:RES (**C**), MAO-A:5-HT (**D**).

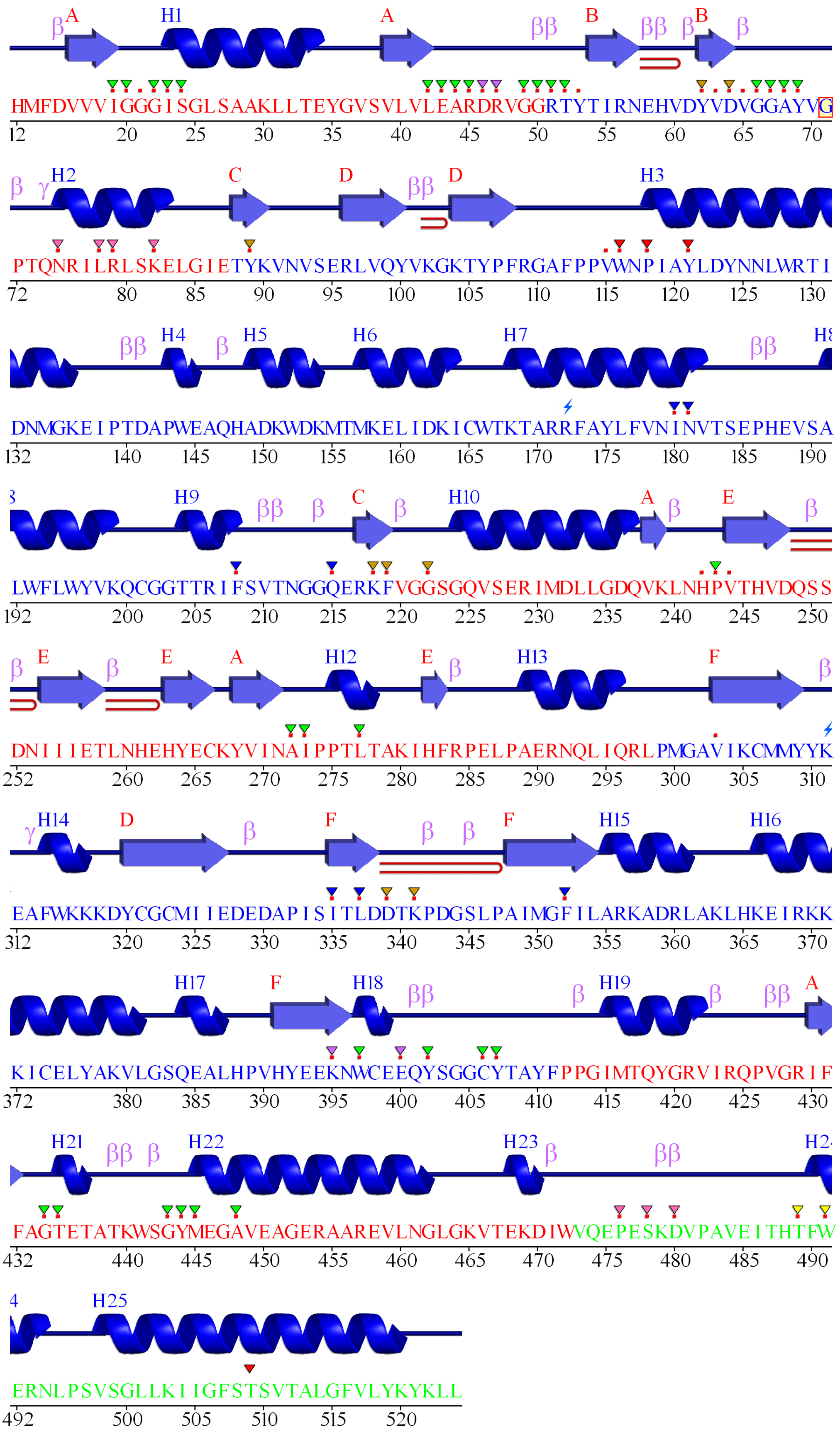

Figure S2. Secondary structure of the MAO-A protein (pdb ID: 2Z5X). Figure is generated using PDBsum web server [1].

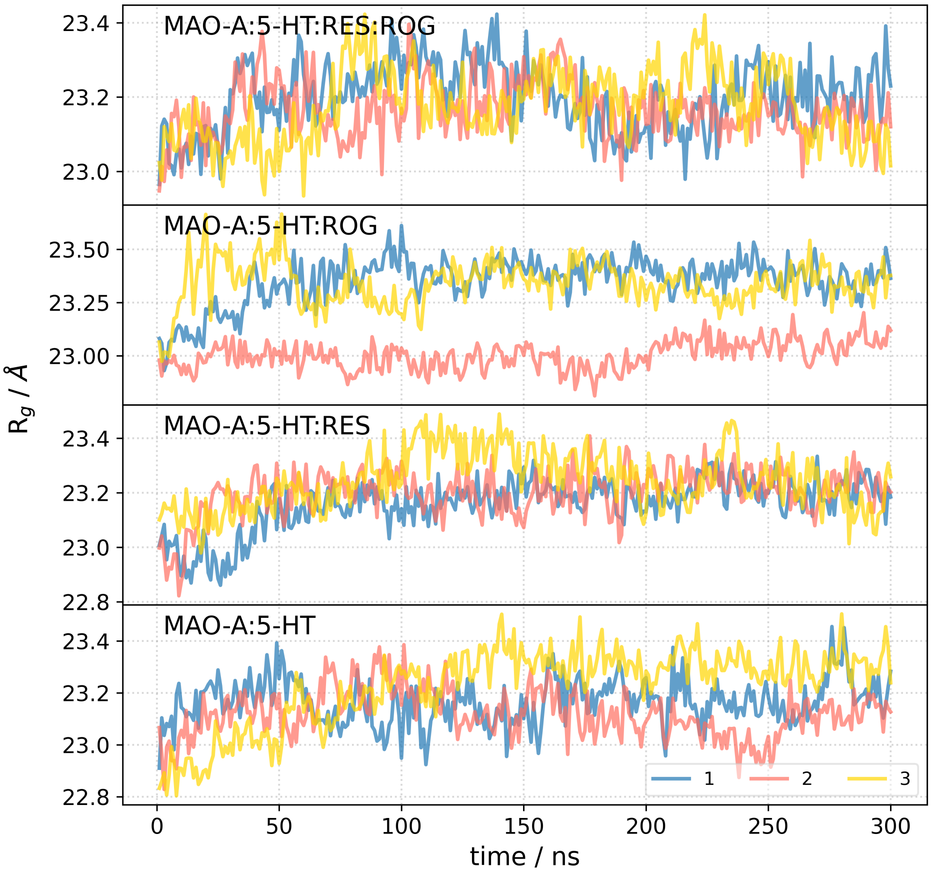

Figure S3. Radius of gyration (R_g) profiles of MAO-A complexes over 300 ns molecular dynamics simulations in triplicate.

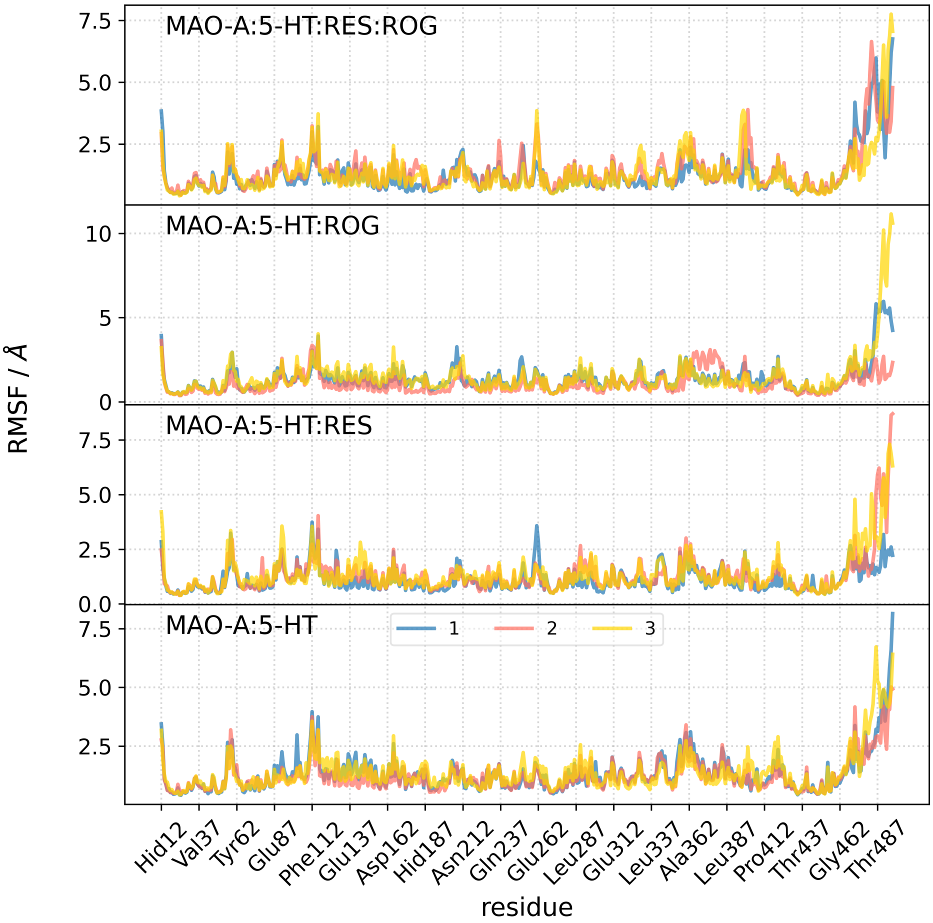

Figure S4. Root mean square fluctuation (RMSF) profiles of MAO-A complexes over 300 ns molecular dynamics simulations in triplicate.

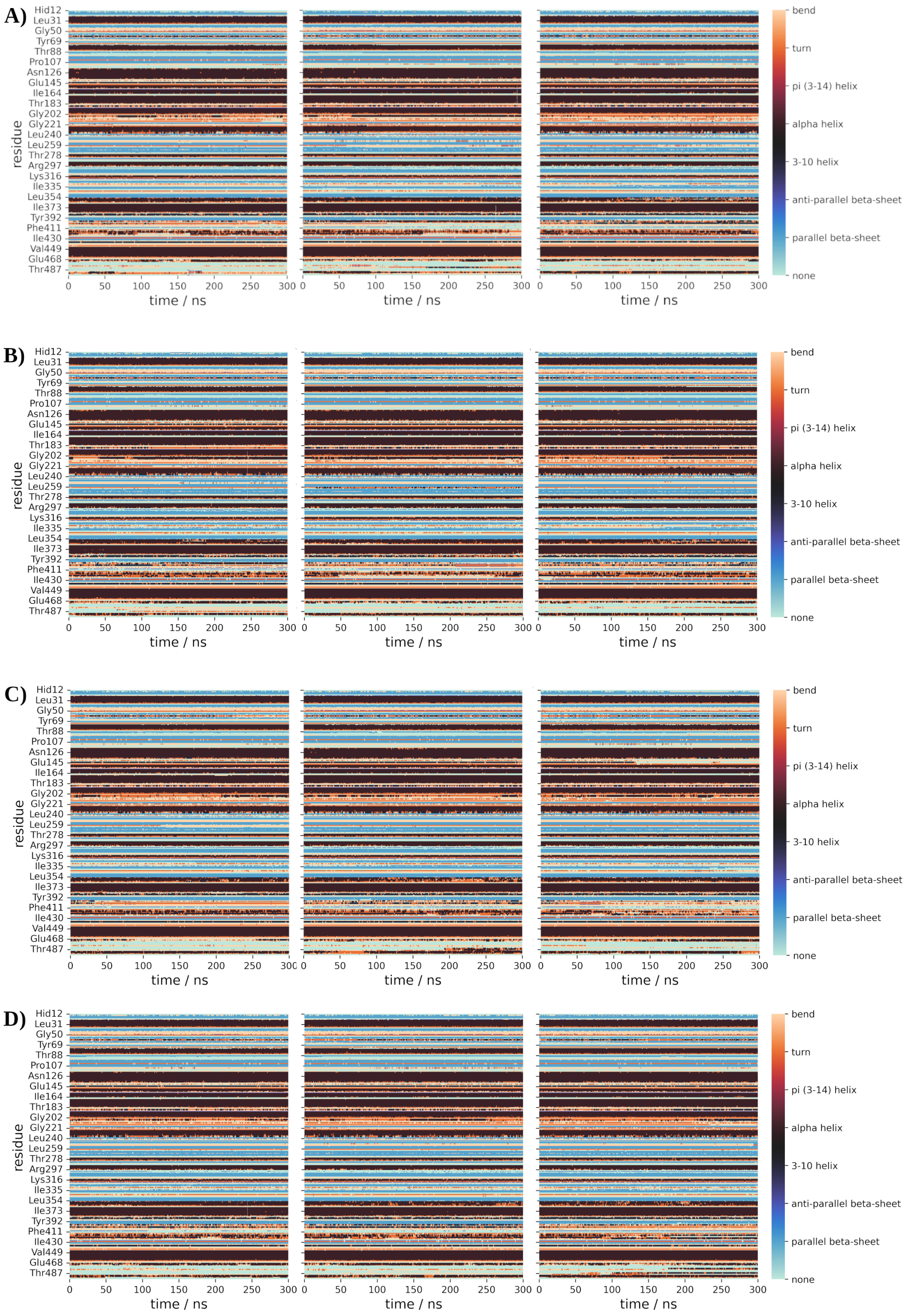

Figure S5. Changes in the secondary structure of the MAO-A:ligand complexes during molecular dynamics simulations in triplicates. MAO-A:5-HT:RES:ROG (**A**), MAO-A:5-HT:ROG (**B**), MAO-A:5-HT:RES (**C**), MAO-A:5-HT (**D**).

References

1. Laskowski, R.A.; Jabłońska, J.; Pravda, L.; Vařeková, R.S.; Thornton, J.M. PDBsum: Structural summaries of PDB entries. *Protein Sci.* **2017**, *27*, 129–134. https://doi.org/10.1002/pro.3289.
